# Composition and Characterization of the Different Lipoproteins in Overweight/Obese Children vs. Normal-Weight Children

**DOI:** 10.3390/biom16070927

**Published:** 2026-06-23

**Authors:** Jose Cuenca-Alcocel, Lorena Villalba-Heredia, Daiana Ibarretxe, Jose A. Casajús, Jose M. Arbonés-Mainar, Pilar Calmarza

**Affiliations:** 1 Clinical Biochemistry Department, Obispo Polanco Hospital, 44002 Teruel, Spain; jose-cuenca1993@hotmail.com; 2 Growth, Exercise, Nutrition and Development (GENUD) Research Group, University of Zaragoza, 50009 Zaragoza, Spain; 3 Faculty of Medicine, Rovira i Virgili University, Vascular Medicine and Metabolism Unit, “Sant Joan” University Hospital, Pere Virgili Institute for Health Research (IISPV), Biomedical Research Networking Center in Diabetes and Associated Metabolic Diseases (CIBERDEM), Avda. Josep Laporte 2, 43204 Reus, Spain; 4 Department of Physiatry and Nursing, Faculty of Health and Sport Sciences, University of Zaragoza, 50009 Zaragoza, Spain; 5 Biomedical Research Center Network for Physiopathology of Obesity and Nutrition (CIBEROBN), Carlos III Health Institute, 28029 Madrid, Spain; 6 Adipocyte and Fat Biology Laboratory (AdipoFat), Transversal Research Unit, Miguel Servet University Hospital, Aragon Health Research Institute, 50009 Zaragoza, Spain; 7 Clinical Biochemistry Department, Miguel Servet University Hospital, 50009 Zaragoza, Spain; 8 Biomedical Research Networking Center for Cardiovascular Diseases (CIBERCV), Carlos III Health Institute, Av. Monforte de Lemos, 3-5. Pavilion 11. Ground Floor, 28029 Madrid, Spain; 9 Commissions on Oxidative Stress and Lipoproteins and Vascular Diseases of the Spanish Society of Laboratory Medicine (SEQCML), 08025 Barcelona, Spain

**Keywords:** overweight/obese children, remnant cholesterol, lipoprotein subclasses

## Abstract

Background: Childhood obesity and overweight have increased considerably in recent years, representing a major global public health problem. This was a comparative study between a group of overweight or obese children and a group of normal-weight children, within an observational setting, performed in a previously studied cohort in which, in the present work, the objective was specifically to evaluate lipoprotein subclasses, particle size, particle number and lipid composition. Methods: We studied the different lipoprotein particles using the Liposcale test. The number of particles of each lipoprotein subclass was quantified by ^1^H-NMR. This method measures the signals emitted by the protons of the terminal methyl group of the four types of lipids present in the lipoprotein particles. Results: It was found that the concentrations of VLDL-C, VLDL-TG, IDL-TG, and HDL-TG, as well as the number of VLDL-Ps and all their subclasses, were statistically higher in the overweight/obese children group. REM-C was also higher in overweight/obese children, and they had a smaller mean LDL-Z. Conclusions: These results support the presence, already in prepubertal childhood, of early metabolic alterations, associated with excess weight, and show that advanced lipoprotein profiling may provide additional information beyond the conventional lipid profile.

## 1. Introduction

Obesity and overweight in childhood and/or adolescence have increased considerably in European countries in recent years [1], currently representing a major global public health problem. In Spain, childhood excess weight remains a significant public health concern. Several studies have consistently shown a high prevalence of overweight and obesity in Spanish children and adolescents, although estimates vary according to age group, region, year of study, and diagnostic criteria [2,3,4]. According to the World Health Organization (WHO), the prevalence of overweight and obesity in Spanish children aged 5–19 years has been estimated at between 31.9% and 37.9% [5]. In a study conducted in 2011, the prevalence of overweight and obesity in Spanish children, aged 8–13 years, was 30.7% and 14.7% respectively [6], and the most recent edition of the ALADINO study (2023) reported a prevalence of overweight and obesity in children aged 6 to 9 years of 20.2% and 15.9%, respectively [7]. Regional differences have also been reported. For example, in Galicia, the prevalence of overweight and obesity in children aged 6–15 years was 24.9% and 8.2%, respectively [3], whereas more recent data from Aragon confirm that childhood excess weight continues to be an important health problem in this region, as well [8]. Altogether, these data indicate that childhood overweight and obesity remain highly prevalent in Spain and continue to represent a major challenge for public health policies.

Childhood obesity can be assessed using different anthropometric methods, depending on age and clinical context. In children under 5 years of age, the WHO recommends weight-for-height or weight-for-length, whereas in older children, obesity is usually defined using age- and sex-specific Body Mass Index (BMI) thresholds, based on growth references [9]. Other measures, such as waist circumference, waist-to-height ratio, mid-upper arm circumference, and skinfold thickness, may provide additional information on body fat distribution, but they are less standardized in pediatric practice [10]. Overall, BMI-based criteria remain the most practical and widely accepted approach for epidemiological studies. In this context, the International Obesity Task Force (IOTF) criteria used in our study seem particularly appropriate, since they provide age- and sex-specific cut-off points linked to the adult BMI thresholds of 25 kg/m^2^ for overweight and 30 kg/m^2^ for obesity.

Childhood obesity is associated with lipid abnormalities [11] and early atherosclerotic changes that may increase the risk of cardiovascular disease (CVD) and glucose metabolism disorders, such as insulin resistance (IR) and type 2 diabetes mellitus (T2DM), can appear, even from early life [12]. Conventional lipid profile analysis usually includes total cholesterol (TC), triglycerides (TG), low-density lipoprotein cholesterol (LDL-C), and high-density lipoprotein cholesterol (HDL-C), but does not provide information on lipoprotein particle number, size, or composition. However, these characteristics are clinically relevant, since patients with the same LDL-C concentration may have different numbers of low-density lipoprotein particles (LDL-Ps) and, therefore, different cardiovascular risk.

In this context, it is important to consider the concept of residual cardiovascular risk, which may persist despite achieving recommended LDL-C targets. A substantial part of this residual lipid-related risk is related to triglyceride-rich lipoproteins and their remnants. These particles, including very low-density lipoprotein particles (VLDL-Ps), triglyceride-rich remnant particles, and intermediate-density lipoprotein particles (IDL-Ps), are atherogenic and can cross the endothelial barrier. Once retained within the arterial wall, their triglyceride component can be hydrolyzed by lipoprotein lipase, releasing free fatty acids and glycerol, and promoting endothelial dysfunction and inflammation. Preclinical, epidemiological, and genetic studies have provided strong evidence for the role of these particles and remnant cholesterol in atherosclerotic cardiovascular disease.

Therefore, analyzing lipoprotein subclasses and their lipid composition may improve the definition of the atherogenic profile and cardiovascular risk [13], even in adolescents and young adults [14]. Several methods are available for this purpose [15,16], among which proton nuclear magnetic resonance (^1^H-NMR) is the highlight, as it allows the simultaneous measurement of the concentration, size, and composition of different lipoprotein subclasses in a single analysis [17].

Although studies in adults with obesity have generally shown consistent alterations in lipoprotein subclasses [18,19], pediatric data remain scarce. Some studies in children with abdominal obesity have reported higher TG concentrations and higher concentrations of all VLDL-P subclasses [20]. With regard to LDL-P subclasses, most studies associate the smallest LDL-P with overweight/obesity, although some discrepancies remain [21,22,23,24,25].

Previous analyses performed in the overweight/obese children cohort of the current study identified differences in conventional lipid and metabolic parameters between overweight/obese and normal-weight children, supporting the presence of an early atherogenic profile in the former group [26]. In the present study, we extend that observation by assessing lipoprotein subclasses and their composition in greater detail using ^1^H-NMR. Moderate increases in triglycerides can already profoundly alter the entire lipoprotein spectrum. Therefore, monitoring them, with advanced tools, such as proton nuclear magnetic resonance (^1^H-NMR), can provide a comprehensive view of residual cardiovascular risk.

Thus, the aim of this study was to analyze the different lipoprotein subclasses (according to their concentration, size, and lipid constituents) in overweight or obese children aged 8–12 years from the Autonomous Community of Aragon, comparing the results obtained with those of a group of normal-weight children of similar ages.

## 2. Materials and Methods

### 2.1. Study Population

This was a comparative study between a group of overweight or obese children and a group of normal-weight children, within an observational setting, conducted in Zaragoza, Aragon, Spain. The study was performed in the same overweight or obese pediatric cohort previously used for other analyses of bone metabolism and conventional lipid and metabolic parameters [26,27], although in the present work, the objective was specifically to evaluate lipoprotein subclasses, particle size, particle number, and lipid composition.

The overweight or obese group consisted of 61 children, selected according to the cut-off points defined by Cole et al. [28] (IOTF criteria). These children were drawn from the Exergames study, carried out at the University of Zaragoza, registered at clinicaltrials.gov (ID number NCT04418713), and approved by the Research Ethics Committee of the Government of Aragon (CEICA, Zaragoza, Spain) on 4 June 2024 (certificate no. 11/2018). The Active Video Games study, also known as “Exergames” [29], aimed to determine the effects of an Active Video Games and Multicomponent Exercise program in overweight or obese prepubertal children.

The normal-weight group was obtained from the controls of the DECOPIN study [30], which aimed to improve the detection of familial hypercholesterolemia in childhood. All children who were overweight or obese according to the IOTF classification criteria [28] were excluded.

**Figure 1:**
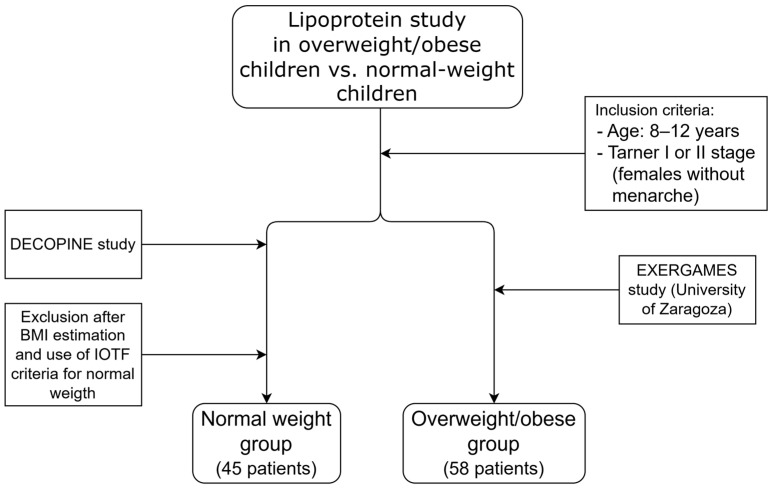
Selection of normal weight and overweight/obese children.

In both groups, children aged 8 to 12 years, who had not yet begun puberty or menarche in girls (Tanner stages I and/or II), did not have pathologies (metabolic or chronic diseases, acute infection, anorexia nervosa) and were not undergoing treatments, that could influence the study parameters, were selected. The selection of the study groups is outlined in Figure 1. After applying these criteria, the normal-weight group consisted of 45 children, while the overweight/obese group included 58 children.

In both cases, a random selection was made from different health centers of healthy children, from similar social classes. Eating habits and physical activity were also assessed in both cases.

First, the parents were informed about the study and provided with all the information in writing, as well as the informed consent document, which was signed by all of them. Next, a brief survey on epidemiological and clinical data was conducted, along with measurements of anthropometric variables: weight, height, and body mass index (BMI). These anthropometric measurements were taken early in the morning on an empty stomach, measuring the height of the children barefoot and with their backs straight. A physical examination and fasting blood tests were also performed to determine the parameters of interest for evaluating lipid metabolism and the metabolic profile. The Z-score for height, weight, and BMI was also calculated for all children, using the 2011 Orbegozo tables [31].

### 2.2. Sample Analysis Procedure

Blood was drawn from children in both groups (overweight/obese children and normal-weight children) early in the morning, after an 8 h overnight fast. The fasting duration, sample processing, and laboratory conditions were identical in both groups. The serum was collected in tubes with a separator gel, which were then centrifuged and aliquoted. These aliquots were frozen and subsequently sent to the Biosfer TestLab laboratory (Reus, Spain), where the profile of the different lipoprotein particles was studied, using the Liposcale^®^ test [17].

In this test, the number of particles of each lipoprotein subclass is quantified by proton nuclear magnetic resonance (^1^H-NMR) spectroscopic analysis. This method measures the signals emitted by the protons of the terminal methyl group of the four types of lipids present in the lipoprotein particles: phospholipids, non-esterified cholesterol, cholesterol esters, and triglycerides. Each signal originates from the sum of the number of terminal methyl groups of the lipids contained in the particle. Triglycerides and cholesterol esters contribute three methyl groups, while phospholipids and non-esterified cholesterol contribute two, respectively.

The different subclasses of lipoprotein particles—VLDL-P, IDL-P, LDL-P, and HDL-P—which have varying sizes, simultaneously emit distinct ^1^H-NMR signals. The amplitudes of these signals (directly proportional to the number of particles in each subclass) can be measured with precision and reproducibility. Thus, using this ^1^H-NMR 2D technology and spectral analysis, the concentration of total VLDL-Ps and LDL-Ps and their different subclasses (large, medium, and small) was obtained, expressed in nmol/L, as well as the concentration of total HDL-Ps and its respective subclasses (large, medium, and small), expressed in µmol/L, the cholesterol and triglyceride content of each lipoprotein particle (VLDL-TG, IDL-TG, LDL-TG, HDL-TG, VLDL-C, IDL-C, LDL-TG and HDL-TG), expressed in mg/dL, and the average size (diameter) of the lipoprotein particles (VLDL-Z, LDL-Z, and HDL-Z), expressed in nanometers. To calculate total cholesterol (TC) and triglycerides (TG), using this technique, the cholesterol and triglycerides of the VLDL-Ps, IDL-Ps, LDL-Ps, and HDL-Ps were summed. Therefore, it is assumed that chylomicrons are not present since the samples were collected after at least 8 h of fasting.

TC = VLDL-C + IDL-C + LDL-C + HDL-C(1)

TG = VLDL-TG + IDL-TG + LDL-TG + HDL-TG(2)

The TG/cholesterol ratio was also calculated in each of the lipoproteins (VLDL-P, IDL-P, LDL-P and HDL-P), as well as the TG/HDL-C ratio, and the non-HDL cholesterol (non-HDL-C) was calculated using the following equation:

non-HDL-C = VLDL-C + IDL-C + LDL-C(3)

And the remnant cholesterol (REM-C) was calculated in the following way:

REM-C = VLDL-C + IDL-C(4)

### 2.3. Statistical Analysis

First, for both the anthropometric and biochemical parameters of the Liposcale^®^ test, the Kolmogorov–Smirnov test with Lilliefors modification (KSL) was used to study the distribution of the quantitative variables, since the number of samples in each group (overweight/obese and normal-weight children) was greater than 30. If the variables followed a normal distribution (KSL, *p* > 0.05), the mean and standard deviation were used for their description; if the quantitative variables did not follow a normal distribution (KSL, *p* ≤ 0.05), the median and interquartile range were used.

For comparisons of the anthropometric and biochemical parameters of the Liposcale^®^ test, between the overweight/obese and normal-weight groups, normally distributed variables were analyzed using Student’s *t*-test when variances were homogeneous and Welch’s test when variances were non-homogeneous, as assessed by Levene’s test. Variables with a non-normal distribution were compared using the Mann–Whitney U test. The comparability of the two study groups was assessed using the same approach for the main anthropometric variables, including age, weight, height, and BMI. Sex distribution between groups was compared using Pearson’s chi-squared test. In cases where statistical significance was less than 0.05, the effect size was also calculated using Cohen’s d for Student’s *t*-test or the Welch test and the Rosenthal Correlation Coefficient for the Mann–Whitney U test. The 95% confidence interval (95% CI) was also calculated for all of them. For Cohen’s d, we considered the effect to be moderate when the coefficient was greater than 0.5 in absolute value and large when it was greater than 0.8 in absolute value. Conversely, for Rosenthal’s correlation, we considered the effect to be moderate when the coefficient was greater than 0.3 and large when it was greater than 0.5.

The dependence of different lipoprotein parameters on age (years), sex (coded as 0 = male and 1 = female), and BMI (BMI Z-score) was studied using multiple linear regression with the input method. The correlation coefficient (R) of the model (which included age, sex, and BMI Z-score) and the adjusted R-squared correlation coefficient were obtained. In addition to age, sex, and BMI Z-score, their respective standardized coefficients (β) and significance (*p*-value) were calculated for each regression. Therefore, we considered that there was an association when the significance was equal to or less than 0.05.

In addition, children were classified according to quartiles (Q1–Q4) of the TG/HDL-C ratio, with cut-off points of 0.96, 1.33, and 1.75, in order to explore the association of this index with lipoprotein parameters (Liposcale^®^ test). Differences across quartiles were evaluated using the appropriate parametric or non-parametric tests, according to variable distribution. First, the Shapiro–Wilk test was used to assess normality within the quartiles, as the number of samples in each quartile was less than 30. In cases where normality was not met in some of the quartiles, the Kruskal–Wallis test was used. In cases where normality was met, Levene’s test was used to verify the homoscedasticity of variances. In the cases where these were comparable, the ANOVA test was applied, and in cases where they were not, Welch’s ANOVA test was applied. When the ANOVA test was significant, post hoc tests were performed using Bonferroni’s test.

The level of statistical significance for all statistical tests used was established at a *p*-value ≤ 0.05. IBM SPSS Statistics version 26.0 was used for the statistical analysis.

## 3. Results

The anthropometric data of the two groups (overweight/obese children and normal-weight children) are shown in Table 1. Regarding age and height, no significant differences were found between the two groups.

As for sex, the proportion of boys and girls in the two groups was also similar (51.7% of boys in the overweight/obese group versus 44.4% of boys in the normal-weight group, *p* = 0.463, Pearson’s chi-squared test).

Furthermore, as expected, both weight and BMI were statistically significantly higher in the overweight/obese group (*p* < 0.001 in both cases), with a large effect size for BMI (|Cohen’s d| > 0.8).

The results for the composition (TG and cholesterol) of the different lipoproteins, as well as the calculated indices and the application of the various statistical tests in the two groups, are shown in Table 2, and those for the concentrations and size of the lipoprotein subclasses are shown in Table 3. It was found that the concentrations of VLDL-C, VLDL-TG, IDL-TG, and HDL-TG, as well as the number of VLDL-Ps and all their subclasses (large, medium, and small), were statistically significantly higher in the overweight/obese children group, while the concentrations of LDL-C and HDL-C, number of LDL-Ps and their medium and small subclasses, and number of HDL-Ps and small subclasses were higher in the normal-weight children. Regarding lipoprotein diameter, the mean LDL-Z was significantly higher in normal-weight children. IDL-C was higher in overweight/obese children, although in this case, the difference did not reach the threshold of statistical significance, despite being close to it (*p* = 0.067). The effect size was moderate in total cholesterol, VLDL-C, LDL-C, HDL-C, total triglycerides, IDL-TG, HDL-T, VLDL-TG/VLDL-C, HDL-TG/HDL-C, TG/HDL-C, REM-C, large and medium VLDL-Ps, medium LDL-Ps, total and small HDL-Ps and LDL-Z. And the effect was large in VLDL-TGs and VLDL-Ps.

**Figure 2:**
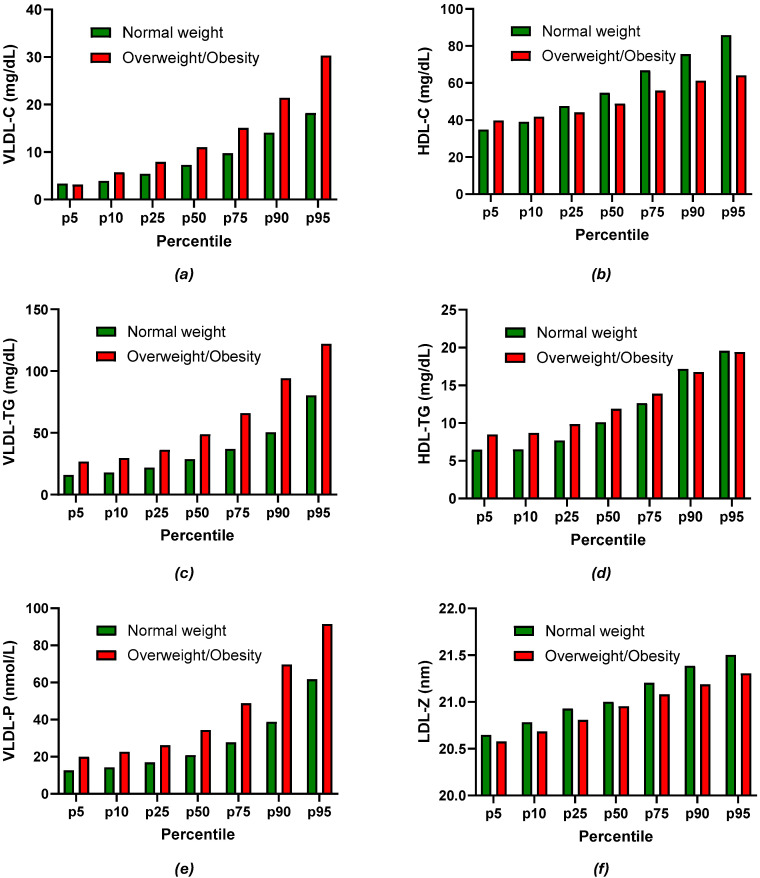
Percentiles of the different lipoprotein parameters in normal-weight children and overweight/obese children: (**a**) VLDL-C; (**b**) HDL-C; (**c**) VLDL-TG; (**d**) HLD-TG; (**e**) VLDL-P; (**f**) LDL-Z.

Furthermore, concerning the calculated indices and parameters, the TG/cholesterol ratio was higher in VLDL-Ps, IDL-Ps, and HDL-Ps in overweight/obese children, and also in LDL-Ps, although no statistically significant differences were found in the last case. Likewise, remnant cholesterol (REM-C) and the TG/HDL-C ratio (*p* < 0.001) were statistically significantly higher in overweight/obese children. The results of the percentiles p5, p10, p25, p50, p75, p90 and p95 calculated in the lipoprotein parameters, in which there were significant differences between the two groups and/or most interesting in this study, are represented in Figure 2.

When studying the multiple linear regression of the aforementioned lipoprotein parameters with sex, age, and BMI (BMI Z-score), we found no association between age and sex with any of the parameters studied, except for Small LDL-P (β = −0.213, *p* = 0.035) and Small HDL-P (β = −0.220, *p* = 0.026) in the case of age, which both decreased with increasing age; and LDL-TG (β = 0.214, *p* = 0.036) and LDL-Z (β = 0.208, *p* = 0.033) in the case of sex, which were higher in girls. In contrast, for the BMI Z score, we did find an association for most of the parameters studied, being positive in the case of VLDL-C, VLDL-TG, IDL-TG, HDL-TG, and VLDL-Ps (total, large, medium and small) and negative in the case of LDL-C, HDL-C, LDL-Ps (total and medium), HDL-Ps (total and small) and LDL-Z. The results of the standardized coefficients along with the significance between the BMI Z score with the parameters studied and R and adjusted R squared of the multiple linear regression for each parameter are shown in Table 4.

**Figure 3:**
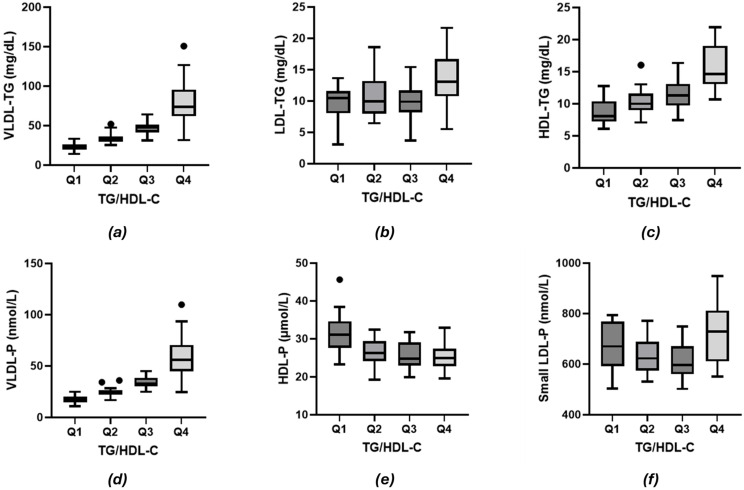
Box plot of TG/HDL-C quartiles with respect to different lipoprotein parameters: (**a**) VLDL-TG; (**b**) LDL-TG; (**c**) HDL-TG; (**d**) VLDL-P; (**e**) HDL-P; (**f**) Small LDL-P.

Moreover, regarding the association of the TG/HDL-C quartiles with the different parameters studied and the Bonferroni post hoc tests, we found that children with a higher TG/HDL-C ratio had a higher BMI, higher concentrations of HDL-TG, VLDL-C, IDL-C, VLDL-TG, IDL-TG, and LDL-TG (in IDL-C and LDL-TG, only Q4 was higher than Q1, Q2, and Q3), VLDL particles (total, large, medium, and small), and small LDL-Ps. On the other hand, children with a higher TG/HDL-C ratio had lower levels of HDL-C and HDL-Ps (total, medium, and small), and smaller LDL-Z particles (Q4 is only smaller than Q2). Those that showed significant differences between quartiles and/or were the most interesting in this study are shown in Figure 3.

## 4. Discussion

In the present study, we found that overweight/obese children showed a more atherogenic lipoprotein profile than normal-weight children, characterized mainly by higher triglyceride enrichment of several lipoprotein fractions, a higher concentration of VLDL-Ps and their subclasses, and a smaller mean LDL-Z. These findings extend our previous analyses in this same cohort, in which overweight/obese children had already shown alterations in conventional lipid and metabolic parameters, including higher triglyceride and insulin concentrations and lower HDL-C and apolipoprotein A1 levels [26], as well as differences in parameters related to bone metabolism [27].

This study represents a significant advance in this field, as knowledge of the different lipoprotein fractions allows us to better assess cardiovascular risk. The number of particles, their size, and their triglyceride and cholesterol composition are relevant determinants of their atherogenic potential, and similar studies to ours conducted in children are very scarce; more specifically, we found none in children aged 9 to 12 years.

In our study, we found that overweight/obese children have higher concentrations of VLDL-TG, IDL-TG, and HDL-TG compared to normal-weight children. We also found a higher TG/cholesterol ratio in all lipoproteins of overweight/obese children compared to normal-weight children (in the case of LDL-TG/LDL-C, the level of statistical significance *p* < 0.05 was not reached).

In this sense, a review of the literature highlights that several studies have found an association between increased HDL-TG and cardiovascular risk, as well as with metabolic disorders and HDL-P dysfunction. For example, a recent cross-sectional study, which included 502 patients with type 2 diabetes or metabolic syndrome, showed that TG-enriched HDL-Ps (HDL-TG) had a strong positive correlation with the fatty liver index (FLI). Their concentration was higher as the components of metabolic syndrome increased, as well as when glucose metabolism worsened [32]. Additionally, it has been found that these lipoprotein particles exhibit a reduction in their antiatherogenic properties the more enriched they are in TG [33,34].

The concentrations of remnant cholesterol (REM-C), as well as the concentrations of both VLDL-C and IDL-C were also higher in overweight/obese children (although in the latter case, statistical significance was not reached).

In this sense, in a study conducted in adults at a Spanish lipid unit, when the study population was subdivided into quartiles, according to their TG concentration, the researchers found that in the highest TG quartile, remnant cholesterol—transported by VLDL and IDL— increased fourfold, reaching up to 30% of total cholesterol [35]. Today, we know that there is a strong causal relationship between elevated remnant cholesterol concentration and a higher risk of CVD, both in primary and secondary prevention. This association is supported by robust genetic research.

Regarding the association between the different parameters and the TG/HDL-C quartiles, our results coincide with those obtained in a study of 592 overweight/obese children and adolescents, where patients in Q4 of TG/HDL-C presented a more atherogenic profile [36]. Thus, in our case, the children included in the highest quartile (Q4) had a higher BMI and a more atherogenic profile than those in Q1. It should be noted that within Q1, 85% were in the normal-weight group, in contrast to Q4, where 80% of the patients were in the overweight/obese group, which further reinforces the atherogenic nature of this group, compared to the normal-weight group.

Furthermore, several studies have demonstrated that it is the VLDL-C, rather than VLDL-TG, that increases the risk of coronary artery disease and acute myocardial infarction (AMI), with the VLDL-C concentration being a very important risk factor for these diseases. It should also be noted that, in our study, the TG/HDL-C ratio was significantly higher in overweight/obese children, and elevated values of this ratio have been reported to be associated with small, dense LDL-Ps (sdLDL) [37,38]. In addition, we found that the average size of lipoproteins, specifically LDL-Z, was smaller in overweight/obese children, further reinforcing the assertion that these children have more atherogenic LDL-Ps [39].

Regarding the different lipoproteins, overweight/obese children presented higher concentrations of VLDL-Ps (total and all its fractions) and lower concentrations of HDL-Ps (total and the small particle subfraction). In this sense, in a recent study by Björnson et al. [40], using genetic data from the UK Biobank, it was discovered that larger VLDL-Ps and remnant particles were more atherogenic than LDL-Ps.

Within the arterial intima, retained VLDL-Ps trigger the infiltration of leukocytes and macrophages; the latter phagocytize the trapped lipoproteins and accumulate their cholesterol content, leading to the formation of foam cells and atherosclerotic plaque [41]. It is important to note that, because VLDL-Ps transport more cholesterol than LDL-Ps, each trapped VLDL-P contributes more cholesterol to the arterial wall intima than each trapped LDL-P.

Moreover, Johansen et al. [42], in a study of 29,039 individuals without AMI at baseline, found that larger VLDL-Ps were associated with higher risk indices than smaller VLDL-Ps, IDL-Ps, and LDL-Ps. However, Wadstrom et al. [43], in a 2025 study, reported that smaller VLDL-P subfractions are associated with a higher risk of CVD, while larger VLDL-P subfractions did not confer as much risk.

In this regard, it is important to know that the two studies evaluate different aspects of VLDL-P subfractions; that is, the number of particles in the first case versus cholesterol content in the second. It is true that, per particle, larger VLDL-Ps contain more cholesterol than smaller ones. However, since smaller VLDL-Ps are more abundant, they contribute more to total cholesterol. This distinction helps explain the differences observed between the two studies. It also appears that smaller particles penetrate the arterial wall more deeply.

Furthermore, we must consider that some studies have shown that large VLDL-Ps are statistically significantly correlated with insulin resistance and the incidence of diabetes, as well as with a fatty liver. And in a study conducted with the same technology in an adult population [44], patients with a higher BMI and premature cardiovascular disease presented a greater quantity of VLDL-Ps (total, large and small) and a lower quantity of LDL-Ps (total, large, medium, and small) compared to healthy individuals, which reinforces the idea that these VLDL-Ps exhibit high atherogenicity.

Based on our results, we can conclude that the overweight/obese children in our study have higher concentrations of VLDL-TG, IDL-TG, and HDL-TG, all of which are more atherogenic, especially HDL-TG, which is also associated with metabolic syndrome, type 2 diabetes, and even fatty liver disease. They also have higher concentrations of cholesterol remnants and VLDL-Ps of all sizes, which have a high atherogenic potential and are also related to insulin resistance and a fatty liver. Finally, they exhibit higher TG/HDL-C ratios, which are associated with sdLDL particles and a smaller average LDL-Z, both characteristics related to more atherogenic LDL-Ps.

All of this indicates that these overweight/obese children have a very atherogenic lipoprotein profile, and this makes it necessary to take precautions, based mainly on a balanced diet, with a limitation of simple sugars and saturated fats, as well as physical exercise and a reduction in abdominal circumference, to improve the present and future cardiovascular health of these children.

Among the limitations of our study, we could point to the small sample size, while its strengths include us having conducted the study in a population where there is practically no literature on the subject. It should also be noted that it would be interesting to increase the sample size, which would allow us to differentiate between sexes and between the groups of overweight and obese children. It would also be very important to add in the analytical element of other biochemical and metabolic tests, to complete the metabolic profile of these children.

## 5. Conclusions

In conclusion, we can say that our group of overweight/obese children aged 8 to 12 years already presents a clearly more atherogenic lipid profile, especially a higher concentration of HDL-TG, VLDL-TG, and VLDL-Ps (in all its subclasses). It is a profile that we would not have detected if we had limited ourselves to studying the classic lipid profile, and makes it necessary to take precautions with these children, based mainly on a balanced diet, as well as physical exercise to reduce their body weight.

## Figures and Tables

**Table 1:** Anthropometric parameters in normal weight and overweight/obese children.

	Overweight/Obesity (n = 58, 30 Males and 28 Females)			Normal Weight (n = 45, 20 Males and 25 Females)			Statistical Significance (Test); Effect Size (95% CI)	Levene’s Test *p*-Value
	Total	Limits	Normality Tests ^3^	Total	Limits	Normality Tests ^3^		
Age (years)	10.17 ± 0.91 ^1^	8.44–12.21	0.200	10 (9–11) ^2^	8–12	0.001	0.800 (Mann–Whitney U test)	
Weight (kg)	55.05 ± 11.43 ^1^	33.40–89.10	0.083	34.74 ± 7.27 ^1^	20.50–54.00	0.200	<0.001 (Welch test); 0.76 (0.66–0.83) ^4^	0.031
Height (cm)	145.38 ± 8.21 ^1^	129–161	0.200	143 ± 10.09 ^1^	122–164	0.200	0.204 (Student’s *t*-test)	0.101
BMI (kg/m^2^)	25.80 ± 3.32 ^1^	20.07–36.00	0.200	16.96 ± 2.00 ^1^	13.49–21.17	0.200	<0.001 (Welch test) −3.72 (−3.72–−2.55) ^4^	0.025
	Median	Limits		Median	Limits			
Z score weight	2.38	0.63–7.25	-	−0.41	−1.77–1.18	-	-	-
Z score height	0.88	−1.15–3.49	-	0.49	−1.82–2.88	-	-	-
Z score BMI	2.70	0.80–6.31	-	−0.65	−1.82–0.60	-	-	-

^1^ Mean ± standard deviation; ^2^ median (Q1–Q3); ^3^ Lillefors-corrected Kolmogorov–Smirnov test; ^4^ Cohen’s d (95% CI).

**Table 2:** Type of distribution and comparative study of the composition of lipoproteins and indices calculated in normal weight and overweight/obese children.

Lipid Parameters	Overweight/Obese		Normal Weight		Statistical Significance (Test) ^6^ Effect Size (95% CI)	Levene’s Test *p*-Value
	Value	Normality Test ^3^	Value	Normality Test ^3^		
Total cholesterol (mg/dL)	182 ± 24 (176–189) ^1^	0.200	196 ± 30 (187–205) ^1^	0.200	**0.017** **(Mann–Whitney U test)** **−0.37 (−0.53–−0.19) ^4^**	0.264
VLDL-C (mg/dL)	11.02; 7.14 (7.92–15.07) ^2^	0.027	7.355; 4.48 (5.42–9.90) ^2^	0.009	**<0.001** **(Mann–Whitney U test)** **0.39 (0.21–0.55) ^4^**	
IDL-C (mg/dL)	6.54 ± 2.92 (5.77–7.31) ^1^	0.200	5.60 ± 2.03 (5.00–6.21) ^1^	0.200	0.067 (Student’s *t*-test)	0.061
LDL-C (mg/dL)	112 ± 18 (108–118) ^1^	0.200	124 ± 20 (118–130) ^1^	0.200	**0.004** **(Student’s *t*-test)** **0.59 (0.19–0.98) ^5^**	0.496
HDL-C (mg/dL)	50.50 ± 7.88 (48.43–52.57) ^1^	0.064	57.47 ± 14.86 (53.06–61.88) ^1^	0.200	**0.005** **(Student’s *t*-test)** **0.63 (0.23–1.02) ^5^**	<0.001
Total triglycerides (mg/dL)	76.4; 35.7 (63.6–99.3) ^2^	0.003	56.7; 21.7 (45.2–66.8) ^2^	<0.001	**<0.001** **(Mann–Whitney U test)** **0.49 (0.33–0.63) ^4^**	
VLDL-TG (mg/dL)	56.15; 29.77 (36.27–66.05) ^2^	0.002	28.69; 15.77 (21.97–37.74) ^2^	<0.001	**<0.001** **(Mann–Whitney U test)** **0.55 (0.40–0.67) ^4^**	
IDL-TG (mg/dL)	8.17 ± 2.19 (7.60–8.75) ^1^	0.200	5.93; 2.23 (5.19–7.42) ^2^	0.007	**<0.001** **(Mann–Whitney U test)** **0.43 (0.27–0.58) ^4^**	
LDL-TG (mg/dL)	10.79 ± 3.15 (9.96–11.62) ^1^	0.200	11.30 ± 3.80 (10.17–12.42) ^1^	0.066	0.463 (Student’s *t*-test)	0.366
HDL-TG (mg/dL)	11.88; 4.03 (9.86–13.88) ^2^	0.009	10.75 ± 3.78 (9.63–11.87) ^1^	0.164	**0.005** **(Mann–Whitney U test)** **0.30 (0.11–0.47) ^4^**	
VLDL-TG/VLDL-C	4.50; 1.13 (3.99–5.11) ^2^	<0.001	4.07 ± 0.74 (3.84–4.29) ^1^	0.200	**<0.001** **(Mann–Whitney U test)** **0.35 (0.16–0.51) ^4^**	
IDL-TG/IDL-C	1.36; 0.37 (1.12–1.50) ^2^	0.007	1.14; 0.28 (1.02–1.31) ^2^	0.004	**0.004** **(Mann–Whitney U test)** **0.28 (0.10–0.45) ^4^**	
LDL-TG/LDL-C	0.094 ± 0.017 (0.090–0.099) ^1^	0.200	0.084; 0.03 (0.073–0.101) ^2^	0.029	0.068 (Mann–Whitney U test)	
HDL-TG/HDL-C	0.23; 0.11 (0.19–0.29) ^2^	0.008	0.17; 0.11 (0.13–0.24) ^2^	<0.001	**<0.001** **(Mann–Whitney U test)** **0.36 (0.18–0.52) ^4^**	
TG/HDL-C	1.59; 0.87 (1.24–2.11) ^2^	<0.001	0.99; 0.77 (0.69–1.46) ^2^	<0.001	**<0.001** **(Mann–Whitney U test)** **0.48 (0.32–0.62) ^4^**	
non-HDL-C	129; 30 (116–147) ^2^	0.003	138 ± 21 (132–144) ^1^	0.200	0.087 (Mann–Whitney U test)	
REM-C	17.42; 9.12 (13.47–22.60) ^2^	0.021	13.41; 6.04 (9.52–15.57) ^2^	0.001	**<0.001** **(Mann–Whitney U test)** **0.35 (0.17–0.51) ^4^**	

^1^ Mean ± standard deviation (95% CI); ^2^ median; interquartile range (Q1–Q3); ^3^ Lillefors-corrected Kolmogorov–Smirnov test; ^4^ Rosenthal Correlation Coefficient (95% CI); ^5^ Cohen’s d (95% CI); ^6^ values in bold indicate statistically significant differences between groups for a 95% confidence level.

**Table 3:** Type of distribution and comparative study of lipoprotein subclass concentration and mean size in normal-weight children and overweight/obese children.

Lipid Parameters	Overweight/Obese		Normal Weight		Statistical Significance (Test) ^6^	Levene’s Test *p*-Value
	Value	Normality Test ^3^	Value	Normality Test ^3^		
VLDL-P (nmol/L)	34.37; 22.55 (26.20–48.76) ^2^	0.001	21.11; 11.03 (17.00–28.02) ^2^	<0.001	**<0.001** **(Mann–Whitney U test)** **0.54 (0.39–0.67) ^4^**	
Large VLDL-P (nmol/L)	1.15 ± 0.46 (1.03–1.27) ^1^	0.061	0.67; 0.41 (0.49–0.90) ^2^	0.002	**<0.001** **(Mann–Whitney U test)** **0.49 (0.33–0.62) ^4^**	
Medium VLDL-P (nmol/L)	3.52; 2.48 (2.75–5.23) ^2^	0.027	2.59; 1.32 (1.74–3.05) ^2^	0.012	**<0.001** **(Mann–Whitney U test)**	
Small VLDL-P (nmol/L)	29.06; 20.47 (22.71–43.18) ^2^	<0.001	17.98; 9.01 (14.88–23.89) ^2^	<0.001	**0.44 (0.26–0.58) ^4^** **<0.001** **(Mann–Whitney U test)** **0.55 (0.40–0.67) ^4^**	
LDL-P (nmol/L)	1119.7 ± 187.5 (1070.3–1169.0) ^1^	0.200	1244.7; 295.9 (1058.6–1354.5) ^2^	0.027	**0.009** **(Mann–Whitney U test)** **−0.25 (−0.06–−0.43) ^4^**	
Large LDL-P (nmol/L)	174.4 ± 24.0 (168.1–180.1) ^1^	0.200	172.6 ± 27.3 (164.5–180.7) ^1^	0.200	0.727 (Student’s *t*-test)	0.190
Medium LDL-P (nmol/L)	298.5 ± 85.9 (275.9–321.1) ^1^	0.200	361.5 ± 95.1 (333.2–389.7) ^1^	0.137	**0.001** **(Student’s *t*-test)** **0.71 (0.30–1.11) ^5^**	0.413
Small LDL-P (nmol/L)	623.2; 121.7 (575.6–697.3) ^2^	0.032	677.1; 136.0 (614.0–750.0) ^2^	0.016	**0.031** **(Mann–Whitney U test)** **−0.20 (−0.01–−0.38) ^4^**	
HDL-P (μmol/L)	26.15 ± 3.48 (25.24–27.07) ^1^	0.200	28.51 ± 5.23 (26.96–30.07) ^1^	0.200	**0.011** **(Welch test)** **0.55 (0.15–0.95) ^5^**	0.028
Large HDL-P (μmol/L)	0.2309 ± 0.0239 (0.2246–0.2372) ^1^	0.099	0.2311 ± 0.0347 (0.2208–0.2414) ^1^	0.143	0.971 (Welch test)	0.031
Medium HDL-P (μmol/L)	8.40; 1.95 (7.58–9.52) ^2^	0.006	9.11 ± 1.80 (8.57–9.64) ^1^	0.157	0.783 (Mann–Whitney U test)	
Small HDL-P (μmol/L)	17.25 ± 2.72 (16.53–17.96) ^1^	0.200	19.17 ± 3.78 (18.05–20.30) ^1^	0.200	**0.003** **(Student’s *t*-test)** **0.60 (0.20–1.00) ^5^**	0.059
VLDL-Z (nm)	42.18 ± 0.21 (42.13–42.24) ^1^	0.200	42.18 ± 0.35 (42.08–42.29) ^1^	0.200	0.988 (Welch test)	<0.001
LDL-Z (nm)	20.95 ± 0.20 (20.89–21.00) ^1^	0.200	21.06 ± 0.23 (20.99–21.13) ^1^	0.200	**0.007** **(Student’s *t*-test)** **0.54 (0.15–0.94) ^5^**	0.316
HDL-Z (nm)	8.22; 0.06 (8.20–8.26) ^2^	0.047	8.24 ± 0.06 (8.22–8.25) ^1^	0.200	0.647 (Mann–Whitney U test)	

^1^ Mean ± standard deviation (95% CI); ^2^ median; interquartile range (Q1–Q3); ^3^ Lillefors-corrected Kolmogorov–Smirnov test; ^4^ Rosenthal Correlation Coefficient (95% CI); ^5^ Cohen’s d (95% CI); ^6^ values in bold indicate statistically significant differences between groups for a 95% confidence level.

**Table 4:** R and adjusted R squared of linear regression multiple and standardized coefficients along with its significance between BMI Z-score with the lipoprotein parameters.

Parameters	R ^1^	Adjusted R Squared ^1^	β ^2^	Significance
VLDL-C	0.346	0.093	**0.349**	**<0.001**
IDL-C	0.187	0.005	0.148	0.143
LDL-C	0.307	0.067	**−0.251**	**0.011**
HDL-C	0.321	0.075	**−0.289**	**0.003**
VLDL-TG	0.439	0.168	**0.441**	**<0.001**
IDL-TG	0.334	0.084	**0.323**	**0.001**
LDL-TG	0.233	0.025	−0.076	0.443
HDL-TG	0.220	0.019	**0.203**	**0.043**
VLDL-P	0.410	0.143	**0.412**	**<0.001**
Large VLDL-P	0.441	0.170	**0.444**	**<0.001**
Medium VLDL-P	0.413	0.145	**0.415**	**<0.001**
Small VLDL-P	0.403	0.137	**0.406**	**<0.001**
LDL-P	0.281	0.051	**−0.211**	**0.033**
Large LDL-P	0.139	−0.011	0.009	0.932
Medium LDL-P	0.365	0.107	**−0.306**	**0.002**
Small LDL-P	0.240	0.029	−0.120	0.225
HDL-P	0.311	0.069	**−0.251**	**0.011**
Large HDL-P	0.194	0.008	−0.021	0.834
Medium HDL-P	0.216	0.018	−0.182	0.070
Small HDL-P	0.326	0.079	**−0.250**	**0.011**
VLDL-Z	0.071	−0.025	0.012	0.903
LDL-Z	0.379	0.117	**−0.273**	**0.004**
HDL-Z	0.225	0.022	−0.086	0.388

^1^ Predictors: constant, age, sex (0: male, 1: female) and BMI Z-score, ^2^ standardized beta coefficients, values in bold indicate statistically significant for a 95% confidence level.

## Data Availability

The raw data supporting the conclusions of this article will be made available by the authors on request.

## Abbreviations

The following abbreviations are used in this manuscript:

^1^H-NMR	Proton nuclear magnetic resonance
AMI	Acute myocardial infarction
β	Standardized coefficient
BMI	Body Mass Index
CVD	Cardiovascular disease
FLI	Fatty liver index
HDL-C	High-density lipoprotein cholesterol
HDL-P	High-density lipoprotein particle
HDL-TG	High-density lipoprotein triglyceride
HDL-Z	High-density lipoprotein size
IDL-C	Intermedium-density lipoprotein cholesterol
IDL-P	Intermedium-density lipoprotein particle
IOTF	International Obesity Task Force
IR	Insulin resistance
KSL	Kolmogorov–Smirnov test with Lilliefors modification
LDL-C	Low-density lipoprotein cholesterol
LDL-P	Low-density lipoprotein particle
LDL-TG	Low-density lipoprotein triglyceride
LDL-Z	Low-density lipoprotein size
R	Correlation coefficient
REM-C	Remaining cholesterol
sdLDL	Small-dense low-density lipoprotein
T2DM	Type 2 diabetes mellitus
TC	Total cholesterol
TG	Triglycerides
VLDL-C	Very-low-density lipoprotein cholesterol
VLDL-P	Very-low-density lipoprotein particle
VLDL-TG	Very-low-density lipoprotein triglyceride
VLDL-Z	Very-low-density lipoprotein size
WHO	World Health Organization
